# The Influence of α-, β-, and γ-Melanocyte Stimulating Hormone on Acetaminophen Induced Liver Lesions in Male CBA Mice

**DOI:** 10.3390/molecules15031232

**Published:** 2010-03-03

**Authors:** Vladimir Blagaić, Karlo Houra, Petra Turčić, Nikola Štambuk, Paško Konjevoda, Alenka Boban-Blagaić, Tomislav Kelava, Marina Kos, Gorana Aralica, Filip Čulo

**Affiliations:** 1 University Hospital “Sveti Duh”, Sveti Duh 64, 10000 Zagreb, Croatia; E-Mail: blagaic@gmail.com (V.B.); 2 University Hospital “Sestre milosrdnice”, Vinogradska cesta 29, 10000 Zagreb, Croatia; E-Mails: khoura@kbsm.hr (K.H.); mackokos@kbsm.hr (M.K.); 3 Faculty of Pharmacy and Biochemistry, University of Zagreb, Domagojeva 2, 10000 Zagreb, Croatia; E-Mail: pturcic@pharma.hr (P.T.); 4 Ruđer Bošković Institute, Bijenička cesta 54, 10002 Zagreb, Croatia; 5 Department of Pharmacology, School of Medicine, University of Zagreb, Šalata 11, 10000 Zagreb, Croatia; E-Mail: abblagaic@mef.hr (A.B.-B.); 6 Department of Physiology, School of Medicine, University of Zagreb, Šalata 3, 10000 Zagreb, Croatia; E-Mails: tkelava@mef.hr (T.K.); fculo@mef.hr (F.Č.); 7 University Hospital Dubrava, Avenija Gojka Šuška 6, 10000 Zagreb, Croatia; E-Mail: garalica@kbd.hr (G.A.)

**Keywords:** melanocortins, alpha-MSH, beta-MSH, gamma-MSH, acetaminophen, liver

## Abstract

Research over the past decade has indicated that melanocortin peptides are potent inhibitors of inflammation and a promising source of new anti-inflammatory and cytoprotective therapies. The purpose of the present paper is to compare protective effects of α-, β-, and γ-melanocyte stimulating hormone on acetaminophen induced liver lesions in male CBA mice. Acetaminophen was applied intragastrically in a dose of 150 mg/kg, and tested substances were applied intraperitoneally 1 hour before acetaminophen. Mice were sacrificed after 24 hours and intensity of liver injury was estimated by measurement of plasma transaminase activity (AST and ALT) and histopathological grading of lesions. It was found that α-, β-, and γ-MSH decrease intensity of lesions by both criteria in a dose-dependent manner.

## 1. Introduction

**Scheme 1:**
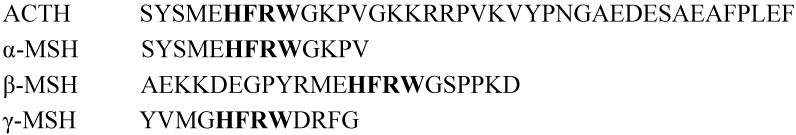
Peptide sequences of ACTH, α-MSH, β-MSH, and γ-MSH. The common amino acid motif sequence (**HFRW**) is depicted by bold characters [3,4].

Acute and chronic inflammatory disorders of the liver are among the greatest challenges of hepatology [1]. Despite some progress in the prevention and pharmacotherapy of such conditions, the development of more effective drugs is still expected [2]. Research over the past decade indicates that melanocortin peptides are potent inhibitors of inflammation and a promising source of new anti-inflammatory and cytoprotective therapies [3,4]. The most investigated peptide from this group is α-melanocyte stimulating hormone (α-MSH), which exhibits anti-inflammatory effects in a different animal models of acute and chronic inflammation [2,3,5,6,7,8,9,10]. α-MSH is generated by proteolysis from larger precursor hormone called proopiomelanocortin (POMC) [3,4]. POMC is the source of other bioactive hormones that share structural homology with α-MSH, including ACTH, β-MSH, and γ-MSH. Peptide sequences of the natural melanocortins are listed in Scheme 1.

Melanocortins exert their effects by activating melanocortin receptors, leading to adenyl cyclase activation and subsequent increase of intracellular cAMP concentration [3,4]. This prevents activation of nuclear factor κB, and subsequently leads to reduction of pro-inflammatory mediators synthesis and adhesion molecules expression [3,4] There are five different melanocortin receptor subtypes (MC-1R to MC-5R) with a sequence homology of 39–61% and a different affinity for the natural melanocortin peptides [3,4] and their receptors (Table 1). β-MSH is an agonist for MC-1R, MC-3R and MC-4R [11,12]. The purpose of the present paper is to compare protective effects of α-, β-, and γ-MSH on acetaminophen induced liver lesions in male CBA mice, a standard *screening* procedure for potential hepatoprotective drugs. Beneficial effects of α-MSH have been observed in models of liver fibrosis and necroinflammation [13,14,15,16]. However, there are no data concerning the role and effects of β- and γ-MSH in necroinflammatory liver lesions. Acetaminophen (APAP) produces liver lesions *via* its reactive metabolite *N*-acetyl-*p*-benzoquinone imine (NAPQI) [17]. Covalent binding of NAPQI leads to disruption of important intracellular proteins and to damage of centrilobular regions of liver after overdose [18]. The loss of cellular integrity is accompanied with the release of intracellular content possessing proinflammatory and hydrolytic capabilities, and a subsequent liberation of numerous cytokine and chemokine mediators from nonparenchymal and nonhepatic cell types [17]. The most prominent proinflammatory mediators in APAP-mediated liver injury are TNF, Fas, interleukin 6, 8, and 11, leukemia inhibitory factor, oncostatin M, and macrophage migration inhibitory factor, MIP-2, MCP-1 [17]. Also, the influx of activated cells such as macrophages, neutrophils, and monocytes contributes to the complexity of events [17]. The final result is the strong inflammatory response of the liver [17]. 

**Table 1:** Melanocortin receptor subtypes and affinity of their ligands [3,4].

*Receptor subtype*	*Affinity of agonists*
MC-1R	α-MSH > ACTH >> γ-MSH
MC-2R	ACTH
MC-3R	γ-MSH = ACTH ≥ α-MSH
MC-4R	α-MSH = ACTH >> γ-MSH
MC-5R	α-MSH ≥ ACTH > γ-MSH

## 2. Results and Discussion

The effects of α-, β-, and γ-MSH in APAP-mediated liver injury were estimated by measurement of plasma transaminase activity and histopathological grading of liver lesions [19,20,21,22,23].

### 2.1. Plasma transaminase activity

The results of aspartate aminotransferase (AST) and alanine aminotransferase (ALT) activity in plasma 24 hours after APAP administration suggest that all tested substances (α-, β-, and γ-MSH) have protective effects (Table 2 and Table 3).

**Table 2:** Aspartate aminotransferase activity (U/L) in plasma 24 h after acetaminophen administration (150 mg/kg i.g.). Tested substances were given intraperitoneally 1 h before acetaminophen. P value is a result of comparison with the control group (Steel’s test).

*Substance*	*Mean*	*SD*	*Median*	*P value*
Control 0.9% NaCl	6767.3	6468.6	5434.0	
α-MSH 6 × 10^-8^ mol/kg*	4880.4	3891.4	3691.0	0.998
α-MSH 3 × 10^-7^ mol/kg*	2613.9	1114.9	2368.5	0.999
α-MSH 6 × 10^-7^ mol/kg*	1418.5	1228.4	905.5	0.398
α-MSH 1.5 × 10^-6^ mol/kg*	539.5	459.6	414.0	0.017
α-MSH 3 × 10^-6^ mol/kg	8845.0	8311.0	7788.0	0.995
β-MSH 5 × 10^-8^ mol/kg	881.9	900.4	669.5	0.145
β-MSH 1 × 10^-7^ mol/kg	182.0	49.3	181.5	0.003
β-MSH 2 × 10^-7^ mol/kg	578.5	268.6	446.0	0.030
β-MSH 4 × 10^-7^ mol/kg	894.4	954.4	459.0	0.070
γ-MSH 5 × 10^-8^ mol/kg	252.1	197.9	173.5	0.004
γ-MSH 1 × 10^-7^ mol/kg	135.3	17.6	138.0	0.003
γ-MSH 2 × 10^-7^ mol/kg	197.4	63.2	175.0	0.003
γ-MSH 4 × 10^-7^ mol/kg	506.3	344.7	473.5	0.023

* Turčić *et al*. [23].

**Table 3:** Alanine aminotransferase activity (U/L) in plasma 24 h after acetaminophen administration (150 mg/kg i.g). Tested substances were given intraperitoneally 1 h before acetaminophen. P value is a result of comparison with the control group (Steel’s test).

*Substance*	*Mean*	*SD*	*Median*	*P value*
Control 0.9% NaCl	9550.0	9213.2	7321.5	
α-MSH 6 × 10^-8^ mol/kg*	8983.7	4654.4	7901.0	0.962
α-MSH 3 × 10^-7^ mol/kg*	4292.0	1422.1	3945.0	0.999
α-MSH 6 × 10^-7^ mol/kg*	2671.3	2235.1	2167.5	0.390
α-MSH 1.5 × 10^-6^ mol/kg*	585.8	1424.1	89.0	0.012
α-MSH 3 × 10^-6^ mol/kg	16853.0	10676.0	18100.0	0.397
β-MSH 5 × 10^-8^ mol/kg	2766.5	2693.6	2119.0	0.270
β-MSH 1 × 10^-7^ mol/kg	55.3	17.7	56.5	0.003
β-MSH 2 × 10^-7^ mol/kg	811.4	440.6	808.5	0.003
β-MSH 4 × 10^-7^ mol/kg	2368.5	3033.9	1322.0	0.041
γ-MSH 5 × 10^-8^ mol/kg	107.8	52.0	82.0	0.003
γ-MSH 1 × 10^-7^ mol/kg	75.8	23.5	82.5	0.003
γ-MSH 2 × 10^-7^ mol/kg	85.1	37.5	80.5	0.003
γ-MSH 4 × 10^-7^ mol/kg	340.4	216.0	340.0	0.003

* Turčić *et al*. [23].

It must be observed that the protective effect of tested substances has the U-shaped dose-response, a common finding in the field of peptide research [24]. The optimal protective dose for the α-MSH was 1.5 × 10^-6^ mol/kg, for β-MSH 1 × 10^-7^ mol/kg, and 1 × 10^-7^ mol/kg for γ-MSH. The increase in dose was followed by diminishing of protective effects. This finding is usually explained as a result of non-specific binding of tested substance to other receptors and molecules.

### 2.2. Histopathological analysis of liver lesions

**Figure 1:**
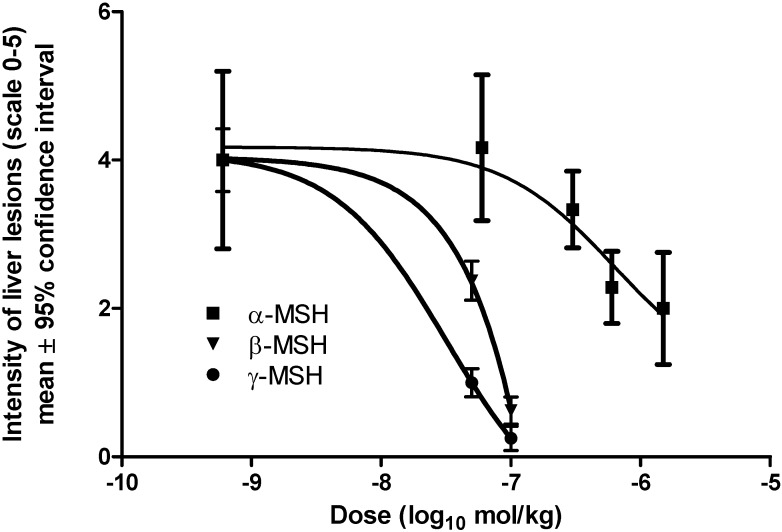
Dose-response curve for α-, β-, and γ-MSH in a prevention of liver necrosis produced by acetaminophen (150 mg/kg i.g.).

Histopathological analysis of liver lesions is considered as the gold standard when defining protective or toxical effects [25]. Two scales were used to grade presence and intensity of liver lesions. The first scale is based on grading of the liver lesions from 0-5, proposed by Silva *et al*. [21]. The second scale is based on the first scale, but simply considers sections with scores ≥3 as significant liver lesions [21,22]. Results of the histopathological grading are presented in Table 4, Table 5, and Figure 1. Both scales confirm results of the transaminase activity, and the U-shaped dose-responses are also observed. 

The optimal protective dose of α-MSH was 1.5 × 10^-6^ mol/kg, for β-MSH 1 × 10^-7^ mol/kg, and 1 × 10^-7^ for γ-MSH. This data confirm the hepatoprotective effects of α-MSH observed in previously published papers [13,14,15,16], but also suggest that potency and efficacy of β- and γ-MSH in this model may even surpass that of α-MSH.

**Table 4:** Intensity of liver lesions 24 h after acetaminophen administration (150 mg/kg i.g.). Tested substances were given intraperitoneally 1 h before acetaminophen. P value is a result of comparison with the control group (Steel’s test).

*Substance*	*Mean*	*SD*	*Median*	*P value*
Control 0.9% NaCl	4.00	1.20	4.5	
α-MSH 6 × 10^-8^ mol/kg*	4.17	0.98	4.5	0.999
α-MSH 3 × 10^-7^ mol/kg*	3.33	0.52	3.0	0.5600
α-MSH 6 × 10^-7^ mol/kg*	2.29	0.49	2.0	0.0320
α-MSH 1.5 × 10^-6^ mol/kg*	2.00	0.76	2.0	0.0178
α-MSH 3 × 10^-6^ mol/kg	5.00	0.0	5.0	0.456
β-MSH 5 × 10^-8^ mol/kg	2.38	0.74	2.5	0.0471
β-MSH 1 × 10^-7^ mol/kg	0.63	0.52	1.0	0.0021
β-MSH 2 × 10^-7^ mol/kg	1.00	1.07	1.0	0.0043
β-MSH 4 × 10^-7^ mol/kg	2.00	1.31	2.0	0.0410
γ-MSH 5 × 10^-8^ mol/kg	1.00	0.54	1.0	0.0023
γ-MSH 1 × 10^-7^ mol/kg	0.25	0.46	0.0	0.0019
γ-MSH 2 × 10^-7^ mol/kg	0.25	0.46	0.0	0.0019
γ-MSH 4 × 10^-7^ mol/kg	0.50	0.76	0.0	0.0026

* Turčić *et al*. [23].

**Table 5:** Number and percentage of animals with histopathology score ≥ 3, 24 h after acetaminophen administration (150 mg/kg i.g.). Tested substances were given intraperitoneally 1 h before acetaminophen. P value is a result of comparison with the control group (Fisher exact probability test).

*Substance*	*Histopathology score* *≥ 3*	*P value*
Control 0.9% NaCl	7/8 (87.5%)	
α-MSH 6 × 10^-8^ mol/kg	6/6 (100%)	0.999
α-MSH 3 × 10^-7^ mol/kg	6/6 (100%)	0.560
α-MSH 6 × 10^-7^ mol/kg	2/7 (28.6%)	0.032
α-MSH 1.5 × 10^-6^ mol/kg	2/8 (25.0%)	0.018
α-MSH 3 × 10^-6^ mol/kg	8/8 (100%)	0.999
β-MSH 5 × 10^-8^ mol/kg	4/8 (50.0%)	0.047
β-MSH 1 × 10^-7^ mol/kg	0/8 (0.00%)	0.002
β-MSH 2 × 10^-7^ mol/kg	0/8 (0.00%)	0.004
β-MSH 4 × 10^-7^ mol/kg	3/8 (37.5%)	0.041
γ-MSH 5 × 10^-8^ mol/kg	0/8 (0.00%)	0.002
γ-MSH 1 × 10^-7^ mol/kg	0/8 (0.00%)	0.002
γ-MSH 2 × 10^-7^ mol/kg	0/8 (0.00%)	0.002
γ-MSH 4 × 10^-7^ mol/kg	0/8 (0.00%)	0.003

α-MSH is the principal agonist with high affinity for all melanocortins receptors (except MC-2R) [3,4]. Affinity measures how strongly a ligand binds to a receptor, but *in vivo* tests determine whether a compound has the desired physiological effects [26]. We observed (Table 2, Table 3, Table 4 and Table 5, Figure 1) that β-MSH and γ-MSH have much stronger hepatoprotective effects then α-MSH, using criteria of potency and efficacy [26,27]. *In vivo* order of β-MSH and γ-MSH potencies may be modified by several confounding factors including differences in metabolic degradation, and action via non-MC receptor mechanisms [28]. MC-3R is thought to be the only melanocortin receptor with sufficient affinity for γ-MSH. It is known to be present in the gut [12], but its presence in the liver of CBA mice remains to be determined. The use of selective antagonists could identify the role of individual melanocortins and their receptor subtypes (MC-1R - MC-5R) in acetaminophen-induced hepatotoxicity. 

## 3. Experimental

### 3.1. Animals

Male CBA mice, bred at Ruđer Bošković Institute, aged 12–16 weeks, were used in the experiment. They were maintained under standard laboratory conditions, with free access to water and commercially available murine food pellets (4RF21, Mucedola, Milan, Italy)

### 3.2. Substances

Pure acetaminophen (APAP) from the Krka pharmaceutical company (Novo Mesto, Slovenia) was used. APAP was dissolved in a warm saline (37 °C) under mild magnetic stirring.

α-MSH (Ac-SYSMEHFRWGKPV-NH_2_, GenScript, USA, purity > 95%) was used in five doses: 6 × 10^-8^ mol/kg (0.1 mg/kg), 3 × 10^-7^ mol/kg (0.5 mg/kg), 6 × 10^-7^ mol/kg (1 mg/kg), 1.5 × 10^-6^ mol/kg (2.5 mg/kg) and 3 × 10^-6^ mol/kg (5 mg/kg).

β-MSH (AEKKDEGPYRMEHFRWGSPPKD, GenScript, USA, purity > 95%) was used in four doses: 5 × 10^-8^ mol/kg (0.125 mg/kg), 1 × 10^-7^ mol/kg (0.25 mg/kg), 2 × 10^-7^ mol/kg (0.5 mg/kg) and 4 × 10^-7^ mol/kg (1 mg/kg).

γ_1_-MSH (YVMGHFRWDRF-NH_2_, GenScript, USA, purity > 95%) was used in four doses: 5 × 10^-8^ mol/kg (0.075 mg/kg), 1 × 10^-7^ mol/kg (0.15 mg/kg), 2 × 10^-7^ mol/kg (0.3 mg/kg) and 4 × 10^-7^ mol/kg (0.6 mg/kg).

Tested substances were dissolved in a warm (37 °C) saline solution.

### 3.3. Treatment regimen

Hepatitis was induced following the procedure described by Guarner *et al*., with slight modifications [19,20,23]. To induce hepatic drug-metabolizing enzymes mice were given phenobarbitone-sodium (Kemika, Zagreb, Croatia) in their drinking water for 7 days in a dose of 0.3 g/L [18,20,23]. Thereafter, mice were fasted overnight and APAP (150 mg/kg) was given intragastrically (i.g.), via a gastric tube, in a volume of 0.5 mL. Mice were re-fed after 4 hours. All tested substances were given intraperitoneally (i.p.) 1 hour before APAP administration, in a volume of 0.2 mL. Control animals were treated with saline (0.9% NaCl). The size of experimental groups was 6-8. Mice that spontaneously died were excluded from histopathological or biochemical analysis.

### 3.4. Plasma transaminase activity

Mice were sacrificed by decapitation 24 hours after APAP application. Heparin (250 U) was given intraperitoneally (i.p.) to each animal 15 minutes before sacrifice, and trunk blood was collected into heparinized tubes. Plasma was separated by centrifugation for 5 min at 8,000 g, and was stored at -20 °C for 24 h before transaminase activity determination. Alanine aminotransferase (ALT) and aspartate aminotransferase (AST) activity was determined by standard laboratory techniques. High standard deviations observed in the measurements of liver enzymes are typical for the experimental model of acetaminophen induced hepatotoxicity in mice, even in highly inbred animals [29,30]. Differences in the absorption and metabolism of acetaminophen between individual animals, changes in temperature, body weight and sampling of blood analytes are some of the contributing factors to high standard deviations of the measurements in small animal models [30]. However, acetaminophen is well suited to satisfy the criteria for an animal model both in terms of clinical relevance, cost, and dose-dependent toxicity [30].

### 3.5. Histopathological analysis of liver lesions

Sections of the liver were fixed in 10% phosphate buffered formalin, embedded in paraffin, sectioned at 4 µm, and stained with hematoxilin and eosin (H&E). Sections were examined by using light microscope at magnification ×100. Two scales were used to grade presence and intensity of lesions [21,22,23]. 

The first scale is based on grades from 0–5:

0. no lesions
1. minimal lesions (individual or a few necrotic cells)
2. mild lesions (10–25% necrotic cells or mild diffuse degenerative lesions)
3. moderate lesions (25–40% necrotic or degenerative cells)
4. marked lesions (40–50% necrotic or degenerative cells)
5. severe lesions (more than 50% necrotic or degenerative cells)

The second scale is based on the first scale, but simply considers sections with scores ≥3 as significant liver lesions [22].

### 3.6. Statistical analysis

Plasma AST and ALT activity, as well as grade of lesions, are expressed as means, medians, and standard deviations. Difference between control and treated groups was determined using Steel's test. Difference between the groups in number of animals with histopathology score ≥ 3 was tested by using Fisher exact probability test. All applied tests were two-tailed. The results were considered significant if *P*-values were ≤ 0.05 [31]. Statistical analysis was made using KyPlot version 4 [32], and dose-response curve was plotted using GraphPad Prism version 5 for Windows [33].

## 4. Conclusions

Collected results of transaminase activity and histopathological grading suggest that α-, β, and γ-melanocyte stimulating hormones prevent APAP-induced liver lesions. The hepatoprotective effect of α-MSH observed in previously published papers was confirmed [13,14,15,16]. However, potency and efficacy of β- and γ-MSH in this model may even surpass α-MSH.
